# Sacubitril/Valsartan as a Therapeutic Tool Across the Range of Heart Failure Phenotypes and Ejection Fraction Spectrum

**DOI:** 10.3389/fphys.2021.652163

**Published:** 2021-08-23

**Authors:** Giovanna Gallo, Massimo Volpe, Allegra Battistoni, Domitilla Russo, Giuliano Tocci, Maria Beatrice Musumeci

**Affiliations:** Cardiology Unit, Department of Clinical and Molecular Medicine, Faculty of Medicine and Psychology, Sant’Andrea Hospital, Sapienza University of Rome, Rome, Italy

**Keywords:** heart failure, left ventricular ejection fraction continuous spectrum, heart failure phenotypes, sacubitril/valsartan, ARNI

## Abstract

Heart failure (HF) is a complex syndrome caused by a variety of structural or functional cardiac abnormalities as a consequence of several involved pathophysiological pathways. In the last decades, left ventricular ejection fraction (LVEF) has represented the principal criterion used to stratify HF, to interpret ventricular function and to identify therapeutic strategies. However, this chimeric parameter oversimplifies the multiple pathways and mechanisms underlying the progression of HF. Indeed, HF should be more appropriately considered as the final stage of multiple disease states, characterized by distinct phenotypes on the basis of key clinical and molecular variables, such as underlying etiologies and conditions, demographic and structural features and specific biomarkers. Accordingly, HF should be viewed as a continuous spectrum in which the specific phenotypes need to be accurately identified with the aim to improve the disease management with a more tailored approach. In such a complex and heterogeneous scenario, the clinical benefits of an angiotensin receptor neprilysin inhibition strategy, namely in the single pill sacubitril/valsartan (S/V), have been shown across the entire HF continuum, representing a fundamental therapeutic strategy, although with different magnitudes depending on the severity and the stage of the clinical syndrome. In this viewpoint paper we have reconsidered the role of S/V in the light of different HF phenotypes and on the basis of HF considered as a whole spectrum.

## Introduction

The current approach to clinical investigation in heart failure (HF) has been substantially focused on the left ventricular ejection fraction (LVEF) viewed as a dichotomous variable to dissect the disease phenotype expression with two distinct categories, namely HF with reduced EF (HFrEF, LVEF < 40%) and HF with preserved EF (HFpEF, LVEF > 50%) (Metra and Teerlink, 2017). This gross subdivision has been instrumental to a simplified approach in randomized controlled trials designed to identify whether a certain pharmacological or non-pharmacological treatment of HF would be beneficial. However, this simplistic approach to the biologic complexity of HF has two major drawbacks: (1) EF is not a categorical dichotomic variable, but rather is a continuous variable which reflects the cardiac pumping properties which, in turn, is the final result of other important factors (inotropism, ventricular synergic contraction, heart rate, pre-load and after-load, ventricular interdependence, myocardial bioenergetics and active myocyte recruitment); (2) while HFrEF mostly reflects its major etiology, i.e., ischemic heart disease, HFpEF is a “garden variety” condition which resembles multiple and very different conditions (aging HF, hypertension mediated LV hypertrophy, diabetic cardiomyopathy, infiltrative cardiomyopathy) which cannot be viewed as a unique disease. Furthermore, it appears not reasonable or biologically plausible to consider patients ranging from 45 to 65% as a whole unique group. In fact, recent European Society of Cardiology Guidelines identified a group with EF ranging from 40 to 49% as HF with mid-range EF (HFmrEF) (Ponikowski et al., 2016).

In this viewpoint paper we have reconsidered the role of sacubitril/valsartan (S/V), an angiotensin receptor blocker/neprilysin inhibitor (ARNI) in the light of different HF phenotypes and on the basis of HF considered as a whole spectrum.

### Heart Failure: From Pathophysiology to Clinical Phenotypes Across a Continuous Spectrum

HF is a progressive syndrome characterized by complex pathophysiological pathways and caused by a variety of structural or functional cardiac abnormalities that lead to elevated intracardiac pressures or to a reduced cardiac output at rest or during stress (Braunwald, 2013).

Cardiac injury with loss of myocyte cells, increased myocardial strain, fibrosis, progressive LV dilatation, change in ventricular shape resulting in cardiac remodeling, leading to increased myocardial oxygen consumption and reduced efficiency of myocardial contraction and arrhythmias are more frequently involved in the development of systolic dysfunction and failure of the pumping properties of the heart (Katz and Rolett, 2016).

On the other hand, vasoconstriction, pro-inflammatory and pro-thrombotic states contribute to impaired ventricular and active atrial relaxation and filling capacities, increased cardiomyocyte passive stiffness, reduced arterial compliance and abnormal ventricular-arterial coupling which finally lead to LV diastolic dysfunction as a final expression (Packer et al., 2020).

Although the above-mentioned pathophysiological mechanisms are frequently shared by the different HF clinical presentations, it may be reasonable to consider HF as the final stage of multiple disease states. A precise determination of distinct phenotypes on the basis of key clinical and molecular variables may help to identify d a more tailored approach rather than a “one-size-fits-all” management strategy (Johnson et al., 2017). Demographic characteristics (such as age, sex, comorbidities, and ethnicity), structural features (such as ventricular wall thickness and cardiac chambers dimensions and compliance) and specific biomarkers of different pathophysiological pathways (such as myocardial stretch, fibrosis, and injury or cardiorenal syndrome) should be combined to improve phenotypic classification and accurately stratify HF patients (Francis et al., 2014; Table 1).

**TABLE 1:** Association between main HF etiologies and LVEF phenotypes.

**Common HF etiologies**	**LVEF**	**Phenotypes**
Coronary artery disease	HFrEF, HFmrEF, less commonly HFpEF	Remodeling characterized by wall thinning and dilatation, fibrosis, and scar; regional wall motion abnormalities. Subendocardial or transmural LGE distribution.
Hypertension	HFpEF, less commonly HFrEF and HFmrEF	Concentric or eccentric hypertrophy. Diastolic dysfunction with increased left filling pressure. Reduced GLS.
Diabetes mellitus	HFpEF, HFrEF, and HFmrEF (particularly in the presence of coronary artery disease)	Thicker LV walls, small indexed LV end-diastolic and end-systolic volumes, high E/e′ ratio, abnormal LV geometry. Endothelial dysfunction, coronary disease, increased fibrosis and deposition of advanced glycation end products.
Aortic stenosis	HFmrEF, HFpEF, less commonly HFrEF	Concentric hypertrophy, impaired LV myocardial deformation, impaired flow reserve, myocardial fibrosis.
Mitral regurgitation	HFrEF, HFmrEF, HFpEF	Increased LA volume and LV preload, eccentric hypertrophy, reduced forward stroke volume, marked LA pressure elevation, pulmonary hypertension.
Idiopathic dilated cardiomyopathy	HFrEF and HFmrEF	LV and/or RV dilatation and dysfunction, diastolic dysfunction with increased left filling pressure.
Hypertrophic cardiomyopathy	More commonly HFpEF	Asymmetric LV hypertrophy, diastolic dysfunction with increased left filling pressure, left atrial dilatation, systolic anterior movement of mitral leaflets with mitral regurgitation. Multi-pattern LGE distribution.
Infection (e.g., myocarditis, Chagas) and systemic immune-mediated disease	More commonly HFrEF and HFmrEF	LV dilatation and systolic dysfunction. Specific immune cell infiltration, myocardial inflammation or diffuse myocardial fibrosis. Subepicardial LGE distribution.
Drugs (e.g., chemotherapy) and toxic agents (e.g., alcohol, cocaine, steroids)	More commonly HFrEF and HFmrEF	LV dilatation and systolic dysfunction, impaired longitudinal and circumferential strain.
Infiltrative myocardial diseases (amyloidosis, hemochromatosis)	More commonly HFpEF	Granular appearance on echocardiography, LV symmetric hypertrophy. RV hypertrophy, increased thickness of the atrio-ventricular valves, thickening of the interatrial septum, small pericardial effusion, restrictive filling Doppler pattern. Diffuse subendocardial LGE distribution.
Chronic kidney disease	HFpEF, HFrEF, and HFmrEF particularly in the presence of coronary artery disease	LV hypertrophy with LV stiffness, diastolic dysfunction with increased left filling pressure, reduced GLS.

*GLS, global longitudinal strain; HFmrEF, heart failure with mid-range ejection fraction; HFpEF, heart failure with preserved ejection fraction; HFrEF, heart failure with reduced ejection fraction; LGE, late gadolinium enhancement; LV, left ventricle; RV, right ventricle.*

Indeed, for too long HF has been arbitrarily stratified only according to LVEF, a criterion chosen mostly to reflect binomial pathophysiological categories, though obviously oversimplifying the multiple pathways and mechanisms underlying the progression of HF. The level of LVEF has been extensively used to provide a simple clinical marker with a high prognostic predictive value, a numerical indicator for therapeutic decision making and finally to distinguish the two main phenotypes (HFrEF and HFpEF) (Triposkiadis et al., 2019). This simple, though not comprehensive, parameter has driven most of evidence based medicine and clinical trials in HF for the last 30 years. On the positive side, this has generated key information for the contemporary management of HF. On the other side, the nature of the information derived from a mere stratification and classification of patients on the basis of EF has probably prevented a more precise therapeutic approach target of the different pathophysiological phenotypes underlying HF in each single individual.

Indeed, the biological basis of a physiological variable together with a growing body of literature speak against the distinction of HF into categories based on the LVEF values and in turn support the hypothesis that HF should be rather viewed as a continuous variable reflecting the whole spectrum of the properties of the LV. Each phenotype and disease trajectory depends on demographic features and risk factors, etiology, functional and structural changes of the heart and therapeutic strategies, which include the potential bidirectional LVEF transition through the recognized classes (De Keulenaer and Brutsaert, 2011; Triposkiadis et al., 2016; Konstam and Abboud, 2017; Lupón et al., 2018). Thus, a dichotomous vision of clinical presentations of HF cannot be adopted anymore in clinical practice to define borders and boundaries of patient classification.

Indeed, although EF remains a key parameter to interpret LV function, it has the disadvantage of being a chimeric index which does not sufficiently represent the major determinants of systolic function including inotropy, lusitropy, preload and afterload, heart rate, and LV synchrony.

Moreover, several studies have demonstrated that systolic and diastolic dysfunction, coronary microvascular dysfunction, endothelial inflammation, oxidative stress, fibrosis, and cardiomyocyte loss may coexist independent of LVEF (Tan et al., 2009; DeVore et al., 2017; von Roeder et al., 2017; Camici et al., 2020). In addition, the detrimental upregulation of renin-angiotensin-aldosterone system (RAAS) has been described in both HF categories although with different degrees of over-activation (Braunwald, 2013; Katz and Rolett, 2016; Ponikowski et al., 2016; Metra and Teerlink, 2017). On the other hand, the natriuretic peptide (NP) system, which may counterbalance the increase of sodium and water reabsorption and the increased vasoconstriction caused by RAAS and SNS, is enhanced across the HF spectrum and may contribute to reduce cardiac hypertrophy and inflammation (McMurray, 2015).

Therefore, it is not surprising that pharmacological strategies able to block the neurohormonal activation, such as inhibitors of the angiotensin converting enzyme (ACEi), angiotensin receptor blockers (ARBs), and mineralocorticoid receptor antagonists (MRA) associated to significant benefits in cardiovascular outcomes (cardiovascular death and re-hospitalization for HF) in HFrEF, repeatedly showed evident trends toward a reduction in morbidity and mortality in other HF subsets characterized by a preserved LVEF (Ponikowski et al., 2016).

In a recent meta-analysis, conducted on 30,882 patients with HFpEF, we showed that treatments based on neurohormonal inhibitors significantly reduce the risk of the primary composite outcome of mortality and hospitalizations for HF and the secondary analysis of HF hospitalizations, without reaching significant benefits on the separate end-point of mortality (Gallo et al., 2020).

On the basis of these pathophysiological considerations, the entire HF spectrum could be better reflected by integrating LVEF with clinical phenotypes.

In this view, the inhibition of the involved neurohormonal systems may represent a fundamental therapeutic strategy through the entire LVEF continuum, although with different magnitudes depending on the severity and the stage of the clinical syndrome.

### Implications of the Unique Mechanism of Action of Sacubitril/Valsartan

As previously mentioned, the pathophysiology of HF is complex, involving the activation of different neuro-hormonal systems such as RAAS. A potential counterbalancing mechanism, the natriuretic peptides (NPs), is also activated in response to increased myocardial wall stress, volume or pressure overload. These latter peptides have recognized diuretic, natriuretic, vasorelaxant, anti-proliferative and anti-hypertrophic properties, and modulate the RAAS (Volpe et al., 2014). However, their role in HF is apparently overridden by the vasoconstriction and sodium retaining actions of RAAS. The action of NPs is mediated through their cell membrane receptors, which are coupled to the particulate guanylate cyclase-cyclic guanosine monophosphate (cGMP) intracellular signaling (Packer et al., 2015). The activation of the effector molecule, protein kinase G (PKG), is linked to the main cardio-protective effects of NPs which potentially inhibit inflammation and leukocyte recruitment, smooth muscle proliferation, vasoconstriction, coronary microvascular impairment, platelet aggregation, fibrosis and hypertrophy. NP cleavage is mostly catalyzed by the neutral endopeptidase neprilysin (NEP) (Bayes-Genis et al., 2016). On the basis of a conceptually meaningful role of NPs catabolism and the theoretically protective significance of its inhibition, development of drugs inhibiting NEP was considered a new attractive strategy to contrast HF development.

The NEP inhibitors were in fact developed to prevent NP degradation and the consequent alteration of the balance between the RAAS and NP system. Since NEP is not specific for NP catabolism and is involved in the degradation of other biological active peptides, such as angiotensin II (Ang II), NEP inhibition alone increases NPs levels, but this effect may be offset by a concomitant rise of Ang II and other peptides. Hence, the concomitant selective blockade of Ang II receptors with an ARB prevents the potential effect of excess Ang II, whereas combining an NEP inhibitor with an ACE-i has been shown to cause unacceptably high rates of angioedema since both NEP and ACE contribute to breakdown of bradykinin (Packer et al., 2002). The composite drug S/V may also reduce the degradation of vasodilator peptides such as bradykinin, substance P, C-type natriuretic peptides, enkephalins, and adrenomedullin resulting in a complex neurohormonal modulation with potentially greater beneficial effects compared with those limited the RAAS inhibition alone (Packer et al., 2002; Volpe et al., 2015; Bayes-Genis et al., 2016; Muiesan et al., 2017). Indeed, urinary cGMP has been found to be elevated after treatment with S/V suggesting that inhibition of NEP together with Ang II receptor blockade may promote the effective binding of NPs to guanylate cyclase-coupled receptors.

The combination of the concomitant inhibition of type 1 Ang II receptor (AT1R) and of NEP achieved with S/V results in a synergistic effect. The AT1R blockade reduces the signal transduction pathways mediated by Gq/11-proteins activating the Ca^2+^ signal, by numerous tyrosine phosphorylated proteins, including the JAK kinase family (JAK2 and Tyk2), and by the phosphokinase- C (PKC)-mediated system, responsible of cellular proliferation, hypertrophy and fibrosis. The increase in NPs levels also produces favorable biological effects mediated by the soluble guanylyl cyclase (sGC)/cGMP pathway (Packer et al., 2002; Volpe et al., 2015; Bayes-Genis et al., 2016; Muiesan et al., 2017).

### The Role of Sacubitril/Valsartan Across LVEF Spectrum

In this complex and heterogeneous scenario, the proposed role of S/V has been tested through the entire HF continuum generating non-univocal data throughout different phenotypes in terms of clinical benefits. The effects on cardiovascular outcomes of S/V have been investigated in a large proof-of-concept study, the PARADIGM-HF (Prospective Comparison of ARNI with ACEI to Determine Impact on Global Mortality and Morbidity in Heart Failure) (McMurray et al., 2014). This double-blind trial randomized 8,442 patients with HFrEF and NYHA (New York Hear Association) class from II to IV to receive S/V or the largely used and validated ACEi enalapril in addition to standard treatment. The trial was stopped before the prespecified term due to outstanding beneficial results obtained in the S/V group. S/V was demonstrated to reduce the risk of the composite primary outcome of cardiovascular death or hospitalization for HF by 20% [hazard ratio 0.80; 95% confidence interval (CI), 0.73–0.87; *P*< 0.001] compared to enalapril. Considering the components of the primary end-point separately, the rate of death from cardiovascular causes and of hospitalization for HF was, respectively, 20 and 21% lower in the group treated with S/V (McMurray et al., 2014). In a *post hoc* analysis of the study, however, S/V maintained its efficacy across the EF spectrum, remaining worthwhile also in patients with a poorer prognosis related to a progressive decline of ventricular function (Solomon et al., 2016a).

Patients treated with lower dosages of S/V had a higher risk of CV events compared to those who maintained the maximal recommended dose of 200 mg twice daily, suggesting a proportional relationship between drug concentrations and achieved benefits (Vardeny et al., 2016).

Subgroup analysis of PARADIGM-HF demonstrated that the benefits of S/V over enalapril were consistent across etiologic categories (infective/viral, alcoholic, valvular, drug-related, peripartum–related), history and timing of previous HF hospitalizations, age categories (also in patients aged ≥ 75 years, although with a higher incidence of hypotension, renal impairment and hyperkalaemia), baseline risk estimated using the MAGGIC (Meta-Analysis Global Group in Chronic Heart Failure) and EMPHASIS-HF (Eplerenone in Mild Patients Hospitalization and Survival Study in Heart Failure) risk scores, the number of signs of congestion (jugular venous distention, edema, rales, and third heart sound) and Charlson co-morbidity index (Jhund et al., 2015; Simpson et al., 2015; Solomon et al., 2016b; Rodil Fraile et al., 2018; Balmforth et al., 2019; Selvaraj et al., 2019). Furthermore, efficacy of S/V was independent from background medications including diuretics, digitalis glycoside and MRA (Okumura et al., 2016).

In this view, S/V may represent a reasonable therapeutic strategy across different HF phenotypes influenced by several determinants, including but not being limited to LVEF.

**FIGURE 1:**
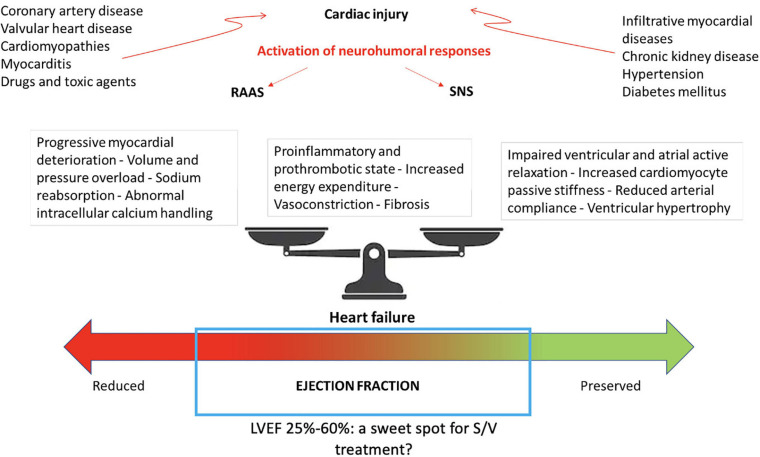
Bidirectional disease trajectories in HF and potential involved pathophysiological mechanisms. LV, left ventricular ejection fraction; RAAS, renin-angiotensin-aldosterone system; SNS, sympathetic nervous system; S/V, sacubitril/valsartan.

On the wave of these exciting results, the benefits of a S/V-based therapeutic strategy based were also investigated in 4,822 patients with LVEF of 45% or higher, randomly assigned to S/V or valsartan in the PARAGON-HF (Angiotensin-Neprilysin Inhibition in Heart Failure with Preserved Ejection Fraction) trial (Solomon et al., 2019). Although with a promising trend toward significance, S/V did not reach the statistical power in the overall study population in the reduction of the composite primary outcome of cardiovascular death and total hospitalization for HF (Solomon et al., 2019). Regarding the secondary end-points, a significant improvement of functional status (NYHA class) and of quality of life (Kansas City Cardiomyopathy Questionnaire) was shown in patients treated with S/V compared to valsartan alone (Solomon et al., 2019). Several mechanisms have been proposed to explain the apparently different results obtained in these two above-mentioned studies. First of all, HFpEF represents a complex and heterogeneous phenotypic set, including patients with several different comorbidities (such as diabetes, atrial fibrillation, chronic kidney disease) and with different clinical characteristics, such as those subjects with wild-type transthyretin (TTR) amyloidosis (Mohammed et al., 2014; Russo et al., 2020) who often remain underdiagnosed or misdiagnosed, being inappropriately enrolled in clinical trials and showing an unsatisfactory response to neurohormonal inhibitors (Maurer et al., 2018). Another explanation has been related to the limited sample and follow-up duration of PARAGON-HF, speculating that a larger trial with a more prolonged observational period might have been able to show significant advantages in HFpEF. Moreover, it has been proposed that the less impressive benefits derived from targeting neprilysin in HFpEF may be a consequence of the lower measured circulating levels of the biological NPs substrate in this subset. A pooled analysis of both trials, including an overall sample of 13,195 subjects (8,399 from PARADIGM-HF and 4,796 from PARAGON-HF) was recently performed aimed to investigate the efficacy of S/V across the ejection fraction spectrum (Solomon et al., 2020). In the overall population, S/V was associated to a significant reduction of the combined end-point of cardiovascular death and first hospitalization for HF (−16%), cardiovascular death (−16%), first hospitalization (−16%), all-cause death (−12%), total HF hospitalizations and cardiovascular death (−18%), and total HF hospitalizations (−19%). The incidence of the primary composite outcome, HF hospitalizations, cardiovascular death, and all-cause mortality was inversely associated to LVEF. However, the most consistent benefits of the treatment with S/V was observed in a LVEF range between 25 and 50% (Solomon et al., 2020). This could correspond to a “sweet spot” for S/V-based therapeutic strategy, thus confirming that ventricular function may represent a significant effect modifier (Figure 1). The data derived by this composite analysis suggest that patients with HFmrEF could be a reasonably successful target for S/V-based treatment, this extending the current recommendations for this pharmacological class (Volpe et al., 2021). Accordingly, considering that the mean LVEF of the population included in the PARAGON-HF was 57% (Solomon et al., 2019), the evidence obtained in this pooled analysis seems to confirm the lack of benefits in a subset of patients with clearly preserved ventricular function. However, the limitations of classifying patients by LVEF cut-offs should be highlighted also in this context (Marwick, 2018). In particular, the beneficial effects of S/V have been demonstrated to extend to higher LVEF in women, suggesting a sex influence, probably related to smaller heart dimensions, to a different adipose-tissue distribution of NPs pharmacokinetic characteristics and possibly to a higher neurohormonal activation (McMurray et al., 2020). Moreover, a recent meta-analysis of 6 studies, with a total of 5,503 patients showed that SV significantly reduced the rate of HF hospitalization (RR, 0.84; 95% CI, 0.77−0.91; *p*< 0.001) and improved the NYHA class (RR, 1.25; 95% CI, 1.10−1.43; *p* = 0.001) in HFmEF and HFpEF patients compared with ACEi and ARB, without a significant increase in side effects (Nie et al., 2020).

According to these results, the US Food and Drug Administration (FDA) has approved an expanded indication for S/V in HFpEF patients, making it the first drug indicated for HF independently from LVEF (Novartis, 2021).

Consistently, future therapeutic strategies in HF, among which S/V represents a first-line choice, should not be based on a single numeric LVEF value, but rather on HF etiology and clinical phenotype (Table 2).

**TABLE 2:** Efficacy of S/V in different categories.

**Benefits of S/V in different clinical subsets**
**LVEF:** S/V has shown efficacy across the LVEF spectrum, despite an increased burden of events in patients with lower LVEF.
**BP:** Also patients with lower SBP generally well tolerate S/V and have the same benefit as patients with higher baseline SBP.
**HF etiology:** The benefit of S/V is consistent across etiologic categories (ischemic, idiopathic, hypertensive or other non-ischemic causes such as infective/viral, alcoholic, valvular, drug-related, peripartum–related).
**Age:** S/V has efficacy across various age categories including aged ≥ 75 years, albeit with a higher incidence of hypotension, renal impairment and hyperkalemia.
**Prior decompensation:** Despite the increased risk associated with more recent hospitalizations, the superiority of S/V does not differ among patients who have never been hospitalized, who have remote HF hospitalizations or who have been more recently hospitalized.
**Concomitant medications:** Efficacy of S/V does not vary according to concomitant medications.
**Renal function:** The effect of S/V is not modified by baseline eGFR, with a lower rate of decrease in eGFR compared to RAS inhibitors.
**CV risk:** The benefit of S/V is maintained across the spectrum of risk estimated with both MAGGIC and other HF-risk scores.
**Diabetes:** S/V is beneficial irrespective of glycemic status, with a reduced incidence of diabetes and a low percentage of subjects requiring oral anti-hyperglycemic therapy or new insulin use.
**Signs of congestion:** S/V reduces CV death and HF hospitalization irrespective of the number of signs of congestion (jugular venous distention, edema, rales, and third heart sound) at baseline and during follow-up.

*BP, blood pressure; CV, cardiovascular; eGFR, estimated glomerular filtration rate; HF, heart failure; LVEF, left ventricular ejection fraction; MAGGIC, Meta-Analysis Global Group in Chronic Heart Failure; MRA, mineralocorticoid receptor antagonists; S/V, sacubitril/valsartan.*

## Conclusion

Lately, the distinction of HF in separate categories according to arbitrary LVEF cut-off seems to be out-of-date, due to the overlapping characteristics of the proposed subgroups and to the complexity of the pathophysiological mechanisms involved in the development of this syndrome, which should be more correctly considered as a unique spectrum composed by different specific phenotypes. In this context, the benefits of a S/V-based strategy have been demonstrated along most of the HF continuum, in which the neurohormonal dysfunction has a pivotal role in the development and progression of the disease.

We still have a long way to go to cover all the unmet needs in HF and a crucial effort should be performed by the medical community to optimize the available pharmacological regimens, particularly selecting a tailored therapeutic approach according to each patient specific phenotypes.

## Author Contributions

GG, DR, AB, and MV contributed to the conception and design, acquisition of data, and analysis and interpretation of data and drafted the article. MV, GT, and MBM contributed to the conception and design and critically revised the manuscript. All authors approved the final version to be published.

## Conflict of Interest

MV reports honoraria for lectures and/or consulting in Advisory Board from Amgen, Astra Zeneca, Daiichi-Sankyo, Menarini Int, MSD, Novartis Pharma, Novo Nordisk, Servier. The remaining authors declare that the research was conducted in the absence of any commercial or financial relationships that could be construed as a potential conflict of interest.

## Publisher’s Note

All claims expressed in this article are solely those of the authors and do not necessarily represent those of their affiliated organizations, or those of the publisher, the editors and the reviewers. Any product that may be evaluated in this article, or claim that may be made by its manufacturer, is not guaranteed or endorsed by the publisher.
